# Ewing Sarcoma of the Pelvis with an Atypical Radiographic Appearance: A Mimicker of Non-malignant Etiologies

**DOI:** 10.7759/cureus.787

**Published:** 2016-09-18

**Authors:** Miguel Flores, Anthony Caram, Edward Derrick, John D Reith, Laura Bancroft, Kurt Scherer

**Affiliations:** 1 Diagnostic Radiology, Florida Hospital-Orlando; 2 College of Medicine, UCF College of Medicine; 3 Pathology, University of Florida

**Keywords:** ewing sarcoma, osteomyelitis, fibrous dysplasia, bone tumor, pediatric, pathology, pediatric tumors, pediatric radiology

## Abstract

Ewing sarcoma (ES) is a primary malignant bone tumor which most commonly arises in children and young adults. The common clinical presentation with ES includes nighttime pain or pain related to activity, though patients may also present with a combination of localized swelling, a palpable mass, pathologic fracture, and constitutional symptoms. Clinical diagnosis may be delayed when a patient presents with clinical or imaging findings that overlap with non-malignant etiologies, such as fibrous dysplasia (FD) or osteomyelitis. Furthermore, multimodality imaging, including computed tomography (CT), magnetic resonance imaging (MRI), and nuclear medicine may prove inconclusive in particular cases. Suspicion for malignancy should not be overlooked. A biopsy must be considered, unless the diagnosis is evident, such as a clinical response to antibiotics in the setting of osteomyelitis.

## Introduction

Ewing sarcoma (ES) is a primary malignant bone tumor that has been classified within a larger group of neoplasms termed Ewing sarcoma family of tumors (ESFT) [1]. ES predominantly arises in children and young adults, second to osteosarcoma in primary malignant bone tumor incidence within the pediatric population. ES represents approximately three percent of all pediatric bone malignancies [1]. A common presentation occurs between the ages of 10 and 19 years old, with the majority of cases presenting between the ages of five and 30 years [2].

Histologically, ES may vary in its degree of neural differentiation, though it most commonly consists of sheets of small, uniform cells with round nuclei, often with an infiltration of the surrounding tissue, hemorrhage and necrosis [2]. The specific progenitor cell for ES is not clear. However, it is hypothesized to be of neural crest or mesenchymal origin [3]. Two sensitive, but not specific, cytological markers for ES include a cluster of differentiation 99 (CD99) and Friend leukemia integration 1 transcription factor (FLI1), with less commonly identified markers including keratin, epithelial membrane antigen (EMA), carcinoembryonic antigen (CEA), and desmin [2-3]. Genetically, ES is characterized by translocations involving the Ewing sarcoma breakpoint region 1 (EWSR1) 22q12 gene locus, with roughly 83% of cases demonstrating a t(11;22)(q24;q12) gene fusion resulting in an EWSR1-FLI1 gene product [4].

A typical clinical presentation with ES includes nighttime pain or pain related to an activity [5]. Patients may also present with a combination of localized swelling, a palpable mass, pathologic fracture, and constitutional symptoms [2, 6]. As part of the initial workup, plain radiographs are often obtained from the affected site. Classically, on plain radiographs, ES demonstrates a permeative or “moth-eaten” appearance, resulting in a layering periosteal bone formation, commonly described as “onion-skin”. This aggressive bone destruction and the periosteal reaction may also be associated with a soft tissue mass [6].

In certain cases, however, ES may not demonstrate radiographically aggressive features. Important considerations during the workup of indeterminate lucent bone lesions in the pediatric and young adult population include fibrous dysplasia (FD) and osteomyelitis, both of which demonstrate clinical and imaging overlap with ES [6-8]. We present the case of a 13-year-old male with progressive left hip pain, indeterminate multi-modality imaging, and a biopsy-proven ES. An informed consent was obtained from the patient referred to in the study.

## Case presentation

A 13-year-old male presented for an evaluation of a progressive left hip pain, worsening over the past five months, with no history of trauma. The patient reported that his hip pain had increased markedly over the past couple of months, resulting in a limp. Furthermore, the patient expressed increasing fatigue, though he denied any night sweats or unintended weight loss. After an evaluation by his primary care physician (PCP), the patient was referred to a pediatric orthopedist, and an imaging of the hip was ordered.

**Figure 1:**
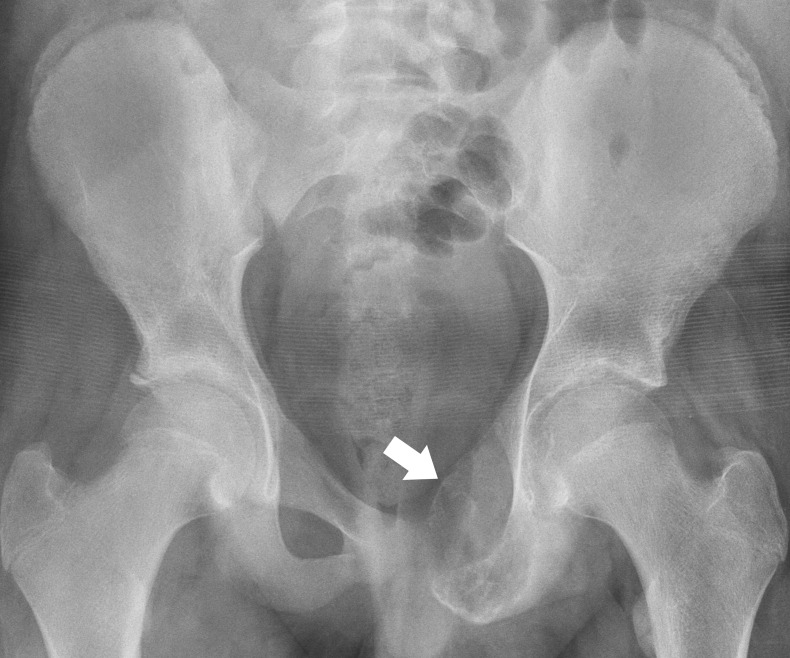
Radiograph of the Pelvis A 13-year-old male with an expansile bony lesion centered within the left pubic bone (white arrow) and involving the left superior pubic ramus, pubic body, and inferior pubic ramus. The lesion did not demonstrate gross cortical disruption, aggressive periosteal reaction, or an association with an overt soft tissue component.

**Figure 2:**
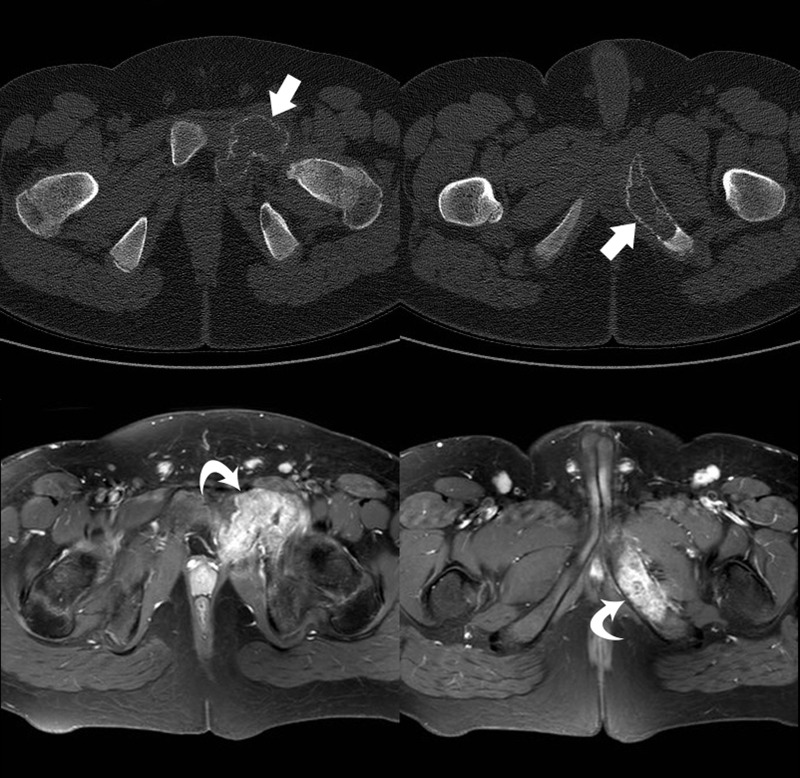
Cross-Sectional Imaging CT axial images (bone window), top row. Gadolinium-enhanced T1-weighted fat-saturated MRI axial images, bottom row. The pelvic lesion demonstrated bony expansion without significant cortical disruption (top row, straight white arrows). The MRI demonstrated heterogeneous enhancement (bottom row, curved white arrows).

**Figure 3:**
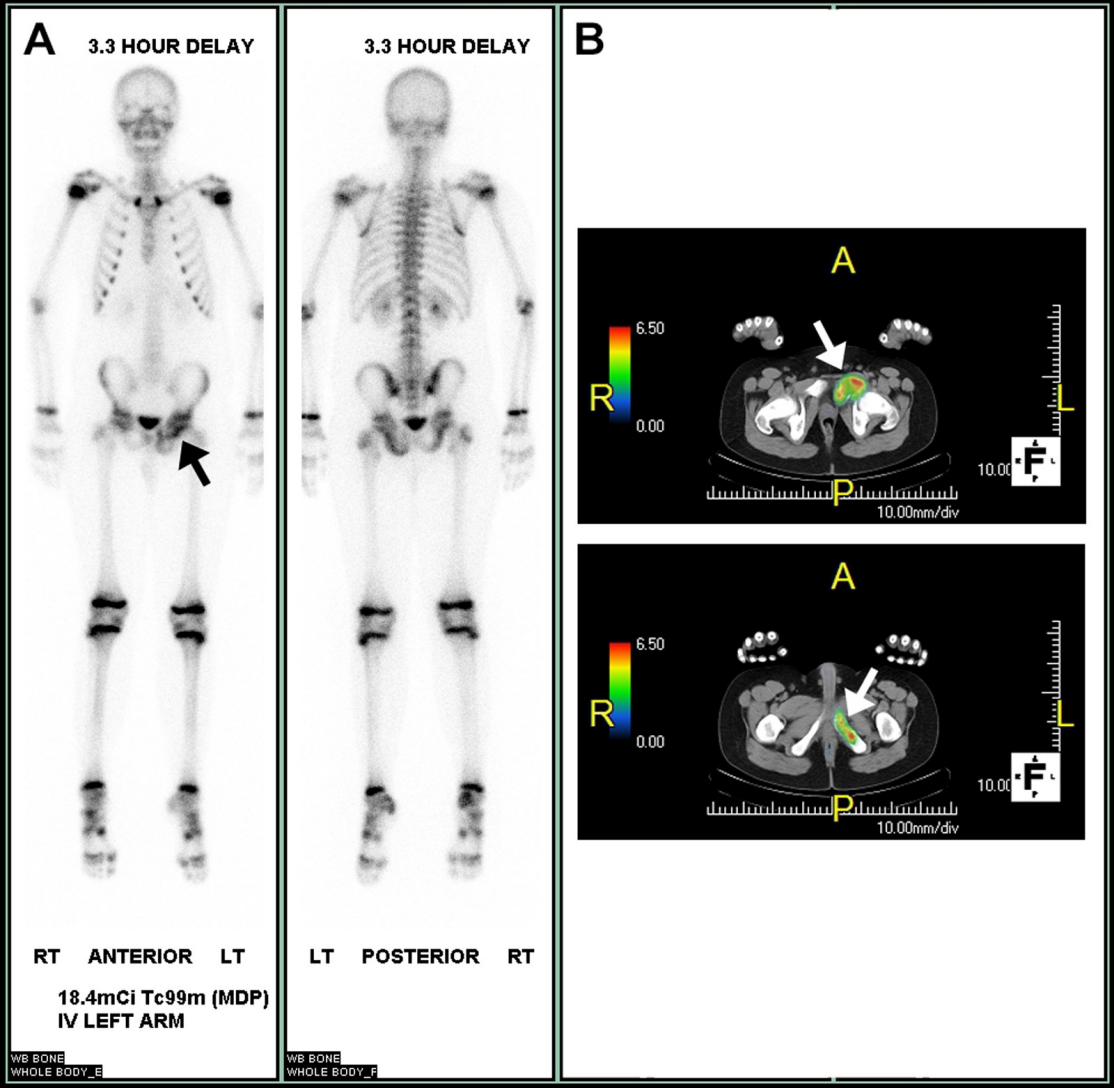
Nuclear Medicine Imaging Technetium 99m-methyl diphosphonate (Tc 99-MDP) Bone scan (3A). 18-Fluoro-deoxyglucose positron emission tomography (FDG-PET), fused axial CT images (3B). Further evaluation with nuclear medicine demonstrated an increased uptake within the left pelvic lesion (3A, black arrow) and associated hypermetabolic activity on FDG-PET (3B, white arrows).

An initial imaging of the left hip consisted of a plain radiograph (Figure 1). The radiograph demonstrated an expansile lesion within the left obturator ring with extension in the left acetabulum, a “ground-glass” appearance, a faintly sclerotic border, and no gross evidence for a soft tissue component. These imaging findings favored a benign etiology. A follow-up imaging for further characterization included computer tomography (CT) and magnetic resonance imaging (MRI) of the left hip (Figure 2). Although the CT provided greater anatomical detail, no definite aggressive features or soft tissue components were appreciated, and a benign etiology was favored. In contrast, the MRI of the left hip demonstrated an expansile lesion involving the left obturator ring and left acetabular wall with a suspicious amount of perilesional edema without definite evidence of a pathologic fracture. Further, imaging included a positron emission tomography (PET)/CT and bone scan, which demonstrated lesional hypermetabolic activity and an increased uptake respectively (Figure 3). These nuclear medicine findings were equivocal considering hypermetabolic activity on PET scan, and an increased uptake on bone scan may be seen with benign lesions, such as FD [9]. The patient subsequently underwent biopsy at an outside institution.

**Figure 4:**
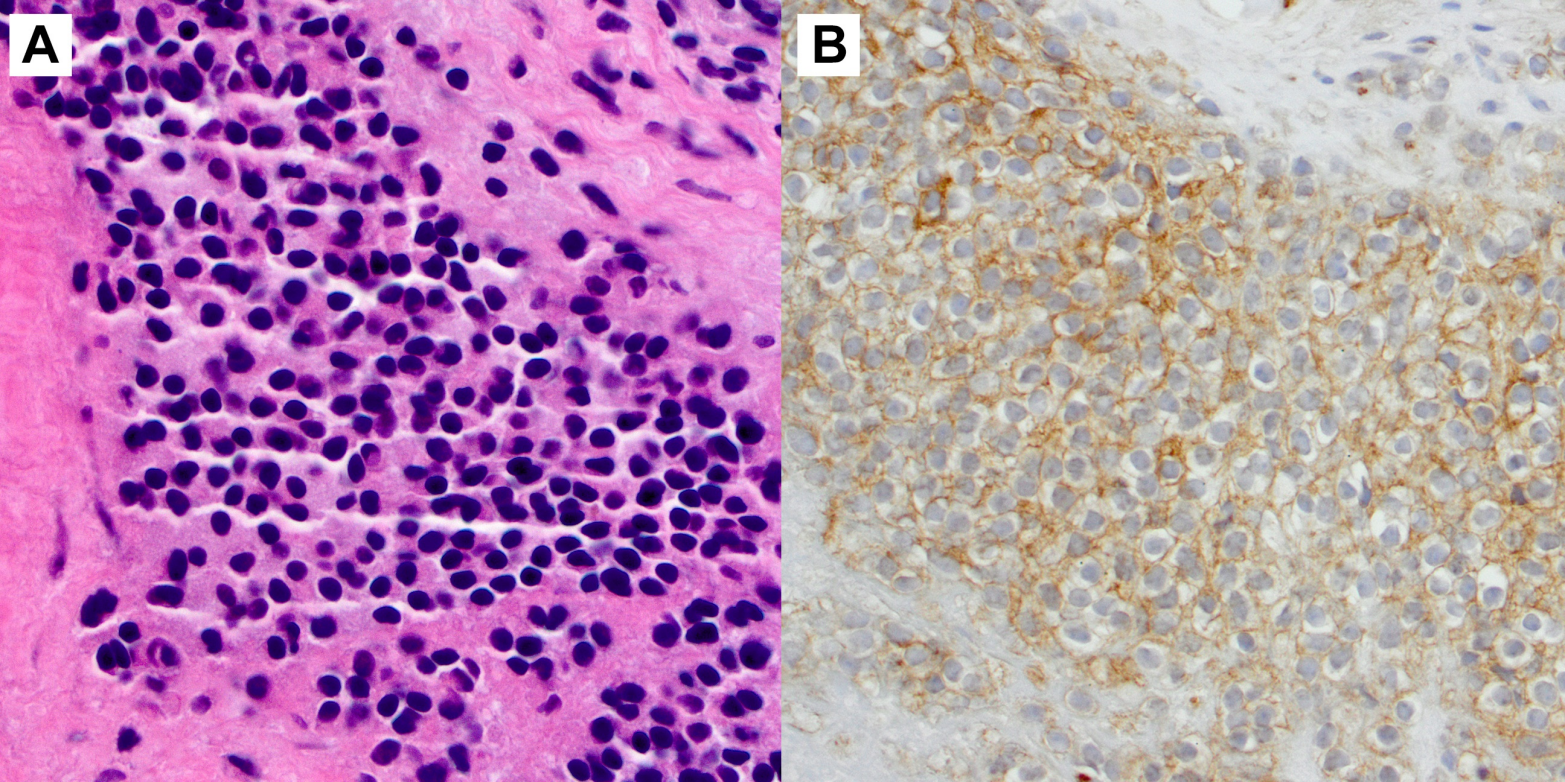
Pathology A biopsy of the pelvic lesion revealed small, primitive cells (4A) infiltrating fibrous tissue and bone (hematoxylin and eosin stain, original magnification x 400). An immunostain for CD99 was strongly immunoreactive in a membranous pattern (4B). A fluorescence in situ hybridization (not pictured) test detected a rearrangement of the EWSR1 gene.

The pathology demonstrated small, primitive cells infiltrating fibrous tissue and bone (Figure 4). An immunohistochemical evaluation revealed tumor cells positive for CD99 and negative for cytokeratin, desmin, myogenin, terminal deoxynucleotidyl transferase (TdT), CD 45, CD3, and CD20, supporting a diagnosis of ES. FISH (fluorescence in situ hybridization) testing was positive for the EWSR1 gene locus rearrangement, further strengthening the diagnosis of ES.

A surgical intervention was offered to the patient and the family. However, after a full consideration of surgical vs. non-surgical treatment options, the decision was made to pursue chemotherapy and radiation. The patient has now completed one full year of curative chemotherapy with vincristine, doxorubicin, and Cytoxan and will begin a short interval of surveillance.

## Discussion

Ewing sarcoma is an infrequent primary bone tumor with a preference for the diaphysis and meta-diaphysis of long bones, accounting for approximately six to eight percent of malignant primary bone tumors in younger patients [6, 8]. The clinical presentation of patients with ES may raise suspicion for malignancy when it involves long bones in a typical location, and is associated with pain and a palpable soft-tissue mass [8]. However, a diagnostic dilemma arises when an ES lesion is present that lacks the characteristically aggressive features or a soft tissue component, and instead, presents with benign findings, such as sclerotic margins, no appreciable periosteal reaction, and no overt soft tissue mass.

A benign presentation on imaging may mimic the appearance of FD, a benign entity characterized by a nonaggressive, geographic lesion with a variable matrix, often associated with bone expansion and remodeling. A review of published literature revealed multiple cases of patients presenting with benign-appearing long bone lesions and characteristic findings of FD with a subsequent pathology diagnostic for ES [6, 8]. Evaluation of an indeterminate lesion with MRI and nuclear medicine may also prove futile, given the overlap of imaging findings between FD and ES. Both FD and ES may demonstrate contrast enhancement on MRI and hypermetabolism on PET scan [9]. Additionally, FD may demonstrate increased activity on gallium and thallium scintigraphy, implying a metastatic focus in patients with synchronous FD and ES [10].

In cases of localized bone pain and swelling with associated fevers and elevated inflammatory markers, osteomyelitis may be initially favored. However, ES may have an identical presentation with fevers and elevated inflammatory markers secondary to tumor necrosis [7]. For this reason, ES should be considered in the differential, when a pediatric patient fails to respond to antibiotics, in a case of presumed osteomyelitis.

## Conclusions

Ewing sarcoma is a primary malignant bone tumor that affects children and young adults. The clinical presentation and imaging findings may have significant overlap with non-malignant etiologies, including fibrous dysplasia and osteomyelitis. In cases of an indeterminate bone lesion with appropriate clinical context, Ewing sarcoma should remain in the differential with the possibility of bone biopsy.
